# Development and application of a non-technical skills coaching intervention framework for surgeons: A pilot quality improvement initiative

**DOI:** 10.1371/journal.pone.0312125

**Published:** 2024-11-08

**Authors:** Marian Obuseh, Nicholas E. Anton, Robin Gardiner, Mengzhou Chen, Shraddhaa Narasimha, Dimitrios Stefanidis, Denny Yu

**Affiliations:** 1 School of Industrial Engineering, Purdue University, West Lafayette, Indiana, United States of America; 2 School of Medicine, Indiana University, Indianapolis, Indiana, United States of America; 3 School of Engineering Education, Purdue University, West Lafayette, Indiana, United States of America; Federal University of Rio Grande do Norte: Universidade Federal do Rio Grande do Norte, BRAZIL

## Abstract

Non-technical skills (NTS) challenges experienced by surgeons may degrade performance, ultimately impacting the safety and quality of care delivered to patients. The objectives of this work were to develop a framework for NTS coaching for surgeons and implement a coaching program utilizing the developed NTS coaching framework. Leveraging adult learning and self-determination theories, a specialty-agnostic NTS coaching framework was developed for individual coaching sessions with robotic surgeons. The framework was used to deliver NTS coaching sessions to robotic surgeons. Surgeon’s robotic procedures were recorded, and expert raters assessed their NTS using the Non-Technical Skills for Surgeons tool. Measures of surgeon satisfaction, learning outcomes, and performance improvement were determined. Cohen’s *d* statistic was used to estimate the effect size of the coaching intervention. To pilot the program, ten robotic-assisted surgeries (five pre-coaching and five post-coaching) were observed from five practicing robotic surgeons who were recruited from a large academic healthcare system. Expert raters’ assessment of surgeons’ NTS revealed several exemplar and non-exemplar behaviors. Surgeons were satisfied with the coaching, rating its quality very highly on all NTS dimensions. On a Likert scale of 1 (strongly disagree) to 5 (strongly agree), surgeons had a stronger agreement that the coaching could improve their situation awareness (4.0±.5) and leadership (4.8±.2) skills compared to their decision making and communication (3.8±.7). From the post-coaching observations, coaching had medium-to-large effect on situation awareness (*d* = .65) and leadership (*d* = .41), small effect on communication and teamwork (*d* = .14), and no effect on decision-making. Overall, the coaching intervention had a medium effect on total NTS (*d* = .33). We presented a quality improvement initiative to enhance the NTS of surgeons by implementing a coaching program that leverages our developed NTS framework. Recognizing the importance of NTS in surgeries, our initiative shows a commitment to continuous improvement of patient safety and quality of care.

## Introduction

Traditionally, adverse surgical outcomes affecting patient safety have been solely attributed to deficiencies in surgeons’ technical skills [1]. However, given recent research findings, more adverse events have been linked to lapses in Non-Technical Skills (NTS) [2, 3]. Specifically, lapses in NTS are responsible for between 43 to 60% of surgical errors [4–6]. While surgeons are extensively trained on technical skills, NTS are not given an adequate emphasis [7]. However, with the increasing adoption of robotic-assisted surgeries (RAS), intraoperative challenges experienced by surgical team members may degrade performance, ultimately impacting patients’ safety [8]. These challenges include new cognitive and learning demands, human-human and human-technology interactions, team communication, and coordination patterns [9]. Consequently, RAS teams are rapidly evolving to meet such intraoperative demands.

In RAS, there is a spatial separation between the surgeon and the rest of the surgical team during robot teleoperation. Hence, NTS like communication and coordination with the rest of the team might be affected [10]. Surgeons have also reported experiencing attentional narrowing with reduced situation awareness while gazing at the console visual displays, teleoperating the robot [11]. Additionally, specific phases of the RAS involve more teamwork than might be expected in an open surgery. For example, robot docking and undocking have a critical influence on team interactions and workflows since the large robot gets moved within the OR [12]. A few studies have specifically evaluated the impact of NTS in RAS [13, 14]. It was found that NTS-related workflow disruptions in RAS were associated with surgical errors [15], increased care duration [16], and threatened patient safety [17].

Surgeons undergo formal training targeted at developing clinical expertise and technical skills. However, surgeons’ NTS have been honed and developed informally and tacitly rather than through any specific professional programs [18]. Quality improvement initiatives targeted at enhancing NTS among robotic surgeons has become a focal point of research and development [19, 20]. Among the various initiatives employed, the concept of coaching emerges as a powerful tool to improve surgeons’ NTS [21, 22]. The absence of personalized feedback to surgeons can limit professional development and higher levels of performance [23, 24]. Personalized NTS feedback recognizes that surgeons might have varying levels of NTS proficiency. Consequently, this offers opportunities for targeted coaching interventions to enhance NTS. Accordingly, the following were the objectives achieved in this study:

1. To design a specialty-agnostic NTS coaching framework for surgeons to:
  - identify exemplar and non-exemplar NTS behaviors
  - deliver targeted and personalized feedback-based coaching sessions to reinforce exemplar behaviors and improve non-exemplar behaviors
2. Apply the framework in a pilot NTS coaching program across different specialties and determine its efficiency in improving robotic surgeons’ NTS

## Materials and methods

### NTS coaching framework design principles and assumptions

In designing the NTS coaching framework, we incorporated critical aspects of both the Adult Learning Theory (ALT) and Self-Determination Theory (SDT). ALT refers to a set of principles and assumptions about how adults learn [25]. SDT is a psychological framework that focuses on the motivation underlying human behavior [26]. It suggests that individuals have innate psychological needs that, when satisfied, contribute to their well-being and motivation. It has been successfully applied in various domains including healthcare [27, 28], psychotherapy [29], and work motivation and management [30, 31]. Within work settings, it has focused on understanding and enhancing motivation, which is important for volitional and sustained engagement in skill development. To achieve positive NTS behavioral changes in this professional group of adults, we leveraged some of the principles and assumptions of both ALT and SDT to design the coaching framework (Table 1).

**Table 1:** Adult learning theory and self-determination theory applied to the design of NTS coaching framework.

**Adult Learning Theory**	
**Assumption**	**Explanation**
Adults are motivated to learn when they have needs and interests that they believe learning satisfies	Attending surgeons bring a wealth of experience to the OR. These experiences are used as a foundation for new learning, relating new information to what they already know, and expanding their learning needs. The framework assumes that surgeons who voluntarily agree to participate in the coaching program recognize that learning might satisfy their growing needs and interests in NTS.
Experience is the richest resource for adult learning	Coaching sessions are based on recent events from surgeons’ recorded procedures. Hence, surgeons learn from their own experiences, as opposed to those of a surgeon peer-coach.
Adults have a need to be self-directing	The role of the NTS coach is not to transmit their knowledge to surgeons and evaluate how they conformed to it. Rather, the coach encourages surgeons to self-identify what they did well (exemplar behaviors) and what they can improve (non-exemplar behaviors).
Effect of individual differences on adult learning	Individual coaching sessions consider the differing skill levels and learning curves of surgeons. Additionally, the coach might need to be flexible to accommodate the surgeon’s schedule and preferred mode of delivery of coaching (online vs in-person).
**Self-Determination Theory**	
**Principle**	**Explanation**
Autonomy	This refers to the desire to be in control of one’s behaviors and goals. Attendings have a greater sense of autonomy than residents during surgeries [32]. This sense of autonomy encourages them to take direct actions that align with their values and results in sustainable changes. This is embodied by a desire for growth in surgical skills.
Intrinsic motivation	Autonomously regulated activities (e.g., skill refinement and mastery, patient-centered care) are intrinsically motivated. Hence, attendings focus primarily on internal sources of motivation such as learning opportunities to improve their NTS.
Competence	The framework does not involve NTS training. Rather, it focuses on NTS coaching. Coaching specifically focuses on improving and refining existing skills, with the goal of empowering coachees to become their own agents of change in their career [33]. This is distinctive from training, which focuses on teaching new skills and concepts [34]. The framework assumes that practicing surgeons have already tacitly developed a level of NTS competence. However, there exists a need for improvement, given the increased attention on its effect on patient safety.
Relatedness	This refers to the fundamental need for social connection and a sense of belonging. During recruitment, potential participants should be informed of the NTS coaching program for robotic surgeons. This relays a sense of community, even though coaching sessions are delivered individually, and participants’ identities are blinded to each other.

### NTS coaching framework for surgeries

The framework proposed in this work utilizes the Non-Technical Skills for Surgeons (NOTSS) [35] system for NTS assessment. The NOTSS system was one of the first behavioral markers for surgeons NTS assessment and describes the main observable behaviors associated with good surgical practice. NOTSS categories include situation awareness, decision making, communication and teamwork, and leadership. Each of the four categories contains three elements each. The observable behaviors are rated on a scale of 1 (performance endangered or potentially endangered patient safety) to 4 (performance was of a consistently high standard and enhanced patient safety). We considered scores of 4 exemplar while scores below 4, non-exemplar [36].

Due to the increased interactions and teamwork involved during the surgical timeout, robot docking and undocking phases, the surgeon’s NTS were evaluated at and within specific timeframes around those events. Using a combined event and time-based approach, the NTS of each surgeon was assessed during the following RAS period: timeout, 10 minutes after timeout end, 10 minutes before robot driving starts through till the end of robot docking, first ten minutes of surgeon on-console, last ten minutes of surgeon on-console, and robot undocking. Procedures are video recorded. Surgeons and/or other members are fitted with wearable microphones to record all verbal interactions. These audio recordings can be synchronized to the video recordings to improve audibility. Surgeon participants are blinded to the specific portions of the procedure they will be assessed on. Trained expert raters utilize NOTSS to assess the surgeons NTS and identify exemplar and non-exemplar behaviors. These are then used to develop the personalized coaching material delivered to each surgeon.

**Fig 1:**
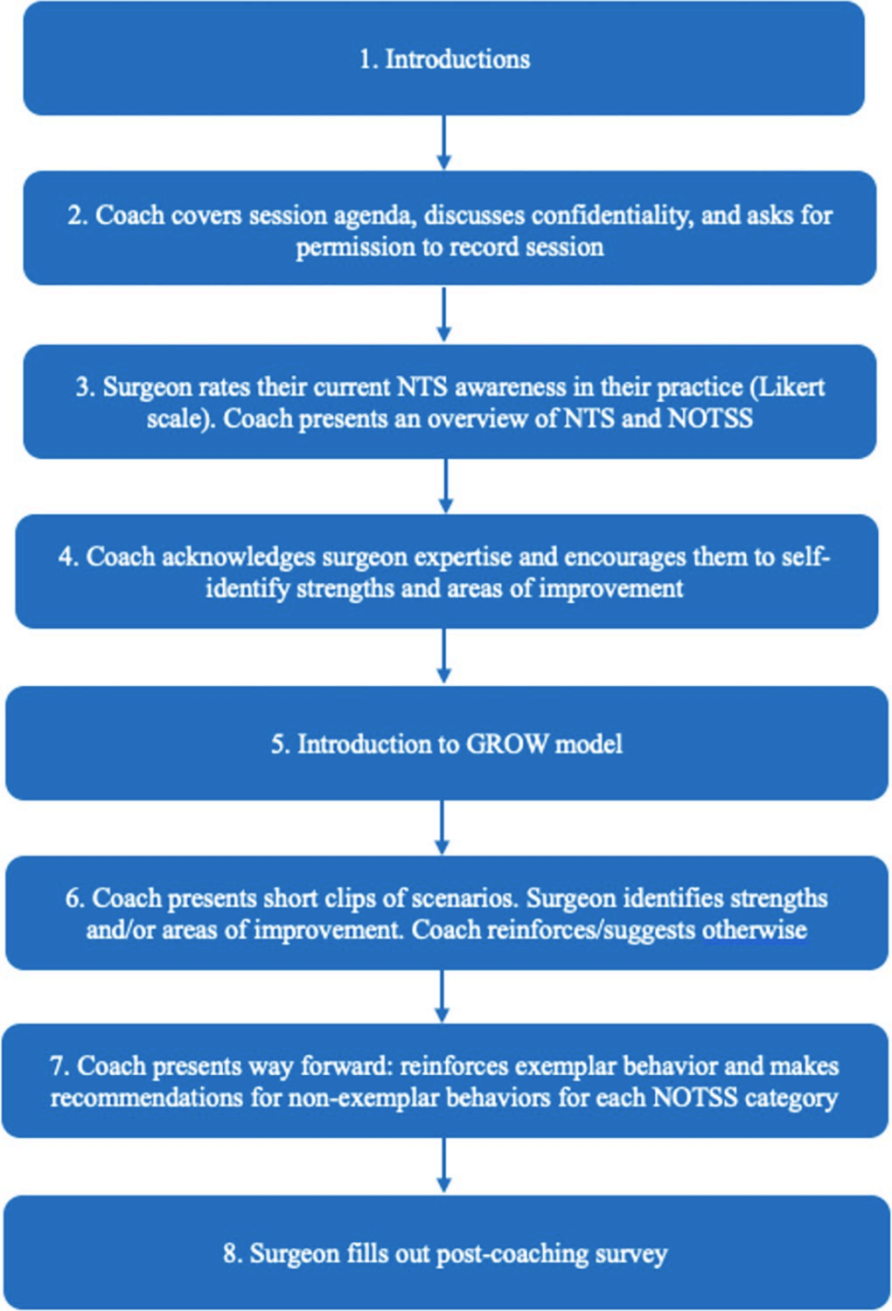
Proposed NTS coaching framework.

Fig 1 summarizes the NTS coaching framework proposed in this study. Research has shown that non-surgeon coaches are more effective than surgeon coaches because surgeon coaches are more likely to prioritize technical skills [21]. Given the association between lapses in NTS and surgical errors, it is imperative that NTS coaching be separated from technical skills coaching. The framework was designed for non-surgeon coaches versed in Human Factors who can direct and focus the coaching strictly on NTS.

The coaching is to be delivered interactively with visuals from a material prepared on a computer presentation program like Microsoft PowerPoint or Keynote. Given the busy schedules of surgeons, coaching sessions should be designed to be brief (25–35 minutes). This also prevents overburdening the demands on the surgeon’s working memory. Below, we review the steps involved in the framework.

#### Step 1

The goal of this step is to establish rapport with the surgeon. The coach introduces themselves, reviews the goal of study, shares their professional background including their experience assessing and enhancing NTS. This step ensures that the surgeon trusts that the coaching will be delivered by an expert who is versed in the field.

#### Step 2

The coach covers the session agenda and discusses confidentiality of the study and the data being collected. If coaching sessions are to be recorded, the coach needs to obtain consent from each surgeon.

#### Step 3

In this step, the coach asks the surgeon to rate their perceived awareness of NTS in their practice. This helps to properly assess the current state of the surgeon’s NTS from the surgeon’s perspective. The coach briefly presents an overview of NTS in surgery and covers each of the four NOTSS categories.

#### Step 4

Here, the coach acknowledges the surgeon’s expertise including their traits of intrinsic-motivation, competency, and sense of autonomy. The coach encourages the surgeon to lean into these attributes to self-identify their strengths and areas of improvement. The coach also set the pace of the session by encouraging the surgeon to view it as a conversation to learn and grow.

#### Step 5

**Fig 2:**
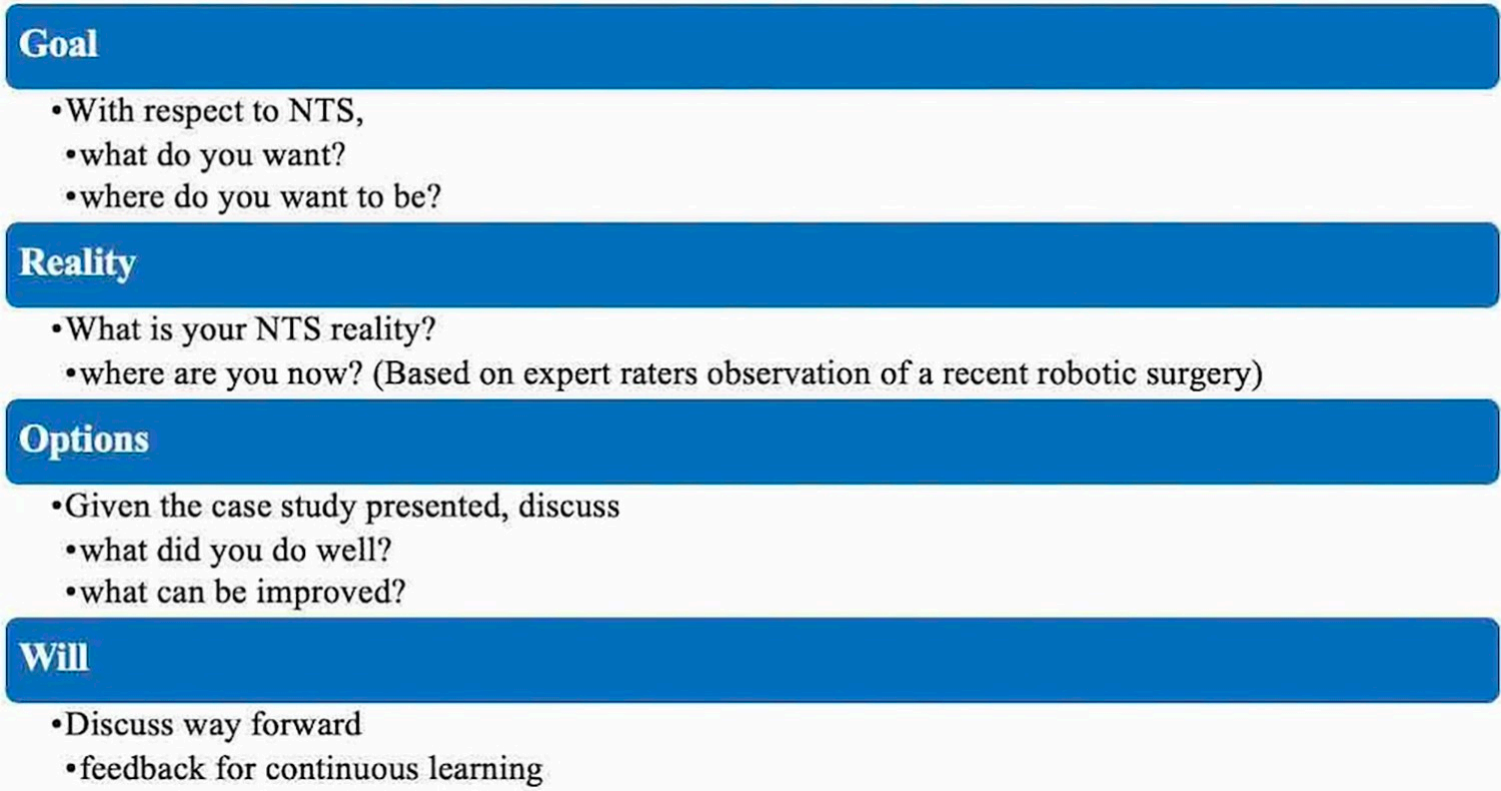
Goal, Reality, Options, and Will (GROW) model applied to non-technical skills coaching sessions.

The surgeon is introduced to the coaching model: Goal, Reality, Options, and Will (GROW) model [37]. Fig 2 shows how the GROW model can be applied during the coaching sessions. A previous study has applied this model to enhance laparoscopic technical skills performance [20].

#### Step 6

In step 6, the coach introduces the surgeon to the case study (specific surgery under review). The next set of pages of the coaching material covers an assessment of the surgeon for each of the four NOTSS categories. Each page contains a one- to three-minute-long video clip with scenarios representing the three elements of each NOTSS category. After each scenario, the coach pauses the video and asks the surgeon to discuss what they did well and what they want to improve with respect to the specific NOTSS category being discussed. This covers the “Options” in the GROW model. The coach either reinforces the surgeon’s comments or presents a differing perspective that the surgeon might not have considered.

#### Step 7

Finally, the coach presents a set of pages covering the “Will” in the GROW model. Each page is a summary of exemplar behaviors the surgeon exhibited and recommendations for improving non-exemplar behaviors. The coach encourages surgeons to continue exhibiting their exemplary behaviors.

#### Step 8

To determine the efficiency of the coaching, the surgeon fills out a survey (Table 2) immediately after the coaching session ends. Depending on the mode of delivery of the session, this step can be administered as a paper or online survey. To ensure openness in responses, the survey is designed such that the identity of each respondent is anonymous. The first set of survey statements aimed at assessing the surgeon’s perception of the coaching session to improve their NTS in the future while the second set of statements asked for the surgeon’s self-assessment of the coaching quality (Table 2). In a previous study, researchers also administered a posttest survey to determine the helpfulness and confidence in NTS training within simulated battlefield first aid scenarios [38].

**Table 2:** Perceptions of coaching to improve future NTS and coaching quality assessment.

**Research Measure: Perceptions of coaching to improve NTS in the future**	
**Survey Statements**	**Response**
The NTS coaching session I just received can improve my **situation awareness** in future procedures	On a Likert scale of 1–5, where: 1 –strongly disagree 2 –somewhat disagree 3 –neither disagree nor agree 4 –somewhat agree 5 –strongly agree
The NTS coaching session I just received can improve my **decision-making** in future procedures	
The NTS coaching session I just received can improve my **communication and teamwork** in future procedures	
The NTS coaching session I just received can improve my **leadership** in future procedures	
**Research Measure: Self-assessment of coaching quality**	
**Survey Statements**	**Response**
Rate the quality of NTS coaching you just received on **situation awareness**	On a Likert scale of 1–5, where: 1 –extremely bad 2 –somewhat bad 3 –neither good nor bad 4 –somewhat good 5 –extremely good
Rate the quality of NTS coaching you just received on **decision-making**	
Rate the quality of NTS coaching you just received on **communication and teamwork**	
Rate the quality of NTS coaching you just received on **leadership**	

### Framework application to NTS coaching program

#### Study participants

The Institutional Review Board of Indiana University approved this study and informed verbal informed consent was obtained from participating surgeons. To pilot the NTS coaching program, five practicing robotic surgeons (3 male, 2 female) across different specialties and with varying levels of surgical experiences were prospectively recruited from an academic healthcare system. Surgeons were recruited between 22 September 2023 and 29 September 2023. The major recruitment criterion was that surgeons perform at least one robotic procedure with Da Vinci Xi model [39] every two weeks. During recruitment, the study coordinator and NTS coach (author MO) reached out individually to potential participants via email, introducing them to the study, goals, timelines, time commitment, and expectations. Surgeons were signed up for the study once they provided verbal informed consent via email, agreeing to participate and commit to the study requirements.

#### Surgical recordings

The robotic procedures of the surgeon participants were video recorded. A lapel microphone was also worn to record their verbal communication. Since surgeons’ NTS will affect and be affected by surgical team members, video recordings included surgical team members (e.g., residents, fellows, anesthesiologists, nurses, and scrubs) and were used in assessing the NTS of only the surgeons. Each surgeon was observed during two procedures and received one coaching session between the procedures. The timelines between observation and coaching varied, depending on the surgeons’ clinical schedules. Coaching sessions were delivered via Zoom within eleven to twenty-one business days after an observation, depending on the surgeon’s availability.

#### Non-technical skills assessment

The study coordinator ensured that no patient identifiers were recorded from the procedures. The video portions considered for NTS assessment were then extracted into a single video file synchronized with the audio recordings. The coordinator then securely distributed the video file to a team of 4–8 trained expert raters with extensive training in human factors, clinical practice, and engineering education for peer review. The NTS coach also served as a rater. The raters individually watched the video and assessed the surgeons’ NTS performance using NOTSS. After the individual assessments, all expert raters met to review their ratings and arrive at a consensus on each surgeon’s NTS. The study coordinator then reviewed both the individual and consensus ratings, which were used in developing the personalized coaching material delivered to each surgeon.

#### NTS coaching

Before starting the study, the NTS coach underwent a peer-to-peer coaching training delivered by a lead Human Factors specialist with over a decade of experience. The coaching materials used in this study were prepared on Microsoft PowerPoint. Coaching sessions were delivered on Zoom using the framework developed in section 2.1.

#### Statistical analysis

The modified Kirkpatrick’s model of training evaluation measures outcomes of coaching on four levels of surgeon satisfaction, learning outcomes, performance improvement, and patient or health outcomes [21, 40]. The effectiveness of the coaching program was determined on measures of surgeon satisfaction, learning outcomes, and performance improvement. Surgeon satisfaction refers to how well the surgeons received the coaching, based on their perceptions and opinions. Learning outcomes refer to any objective change in NTS level during the intervention among surgeons. Both surgeon satisfaction and learning outcomes were determined from the post-coaching survey responses. The performance improvement measure assesses if learning influenced post-coaching NTS behavior. After coaching all the surgeons in the pilot program, mean and standard error of means (SEM) scores for each NOTSS category and overall NTS (before and after coaching) was determined. Effect size was calculated using Cohen’s statistic (*d*) to determine the magnitude of the effect of the NTS coaching intervention [41]. This value reflects if the magnitude of the true underlying difference between NTS scores before and after coaching intervention is practically important [42]. The cutoff for effect sizes were 0.2 for small, 0.5 for medium, and 0.8 for large effect sizes.

## Results

### Coaching program summary

Ten robotic cases were observed across general, urology and bariatric specialties between 2022 and 2023. Mean and SEM for surgeons’ years of experience was 6.8±1.7 years. Mean and SEM for surgeon NTS awareness score before receiving the coaching intervention was 3.2±0.4.

### Evaluation outcomes

**Fig 3:**
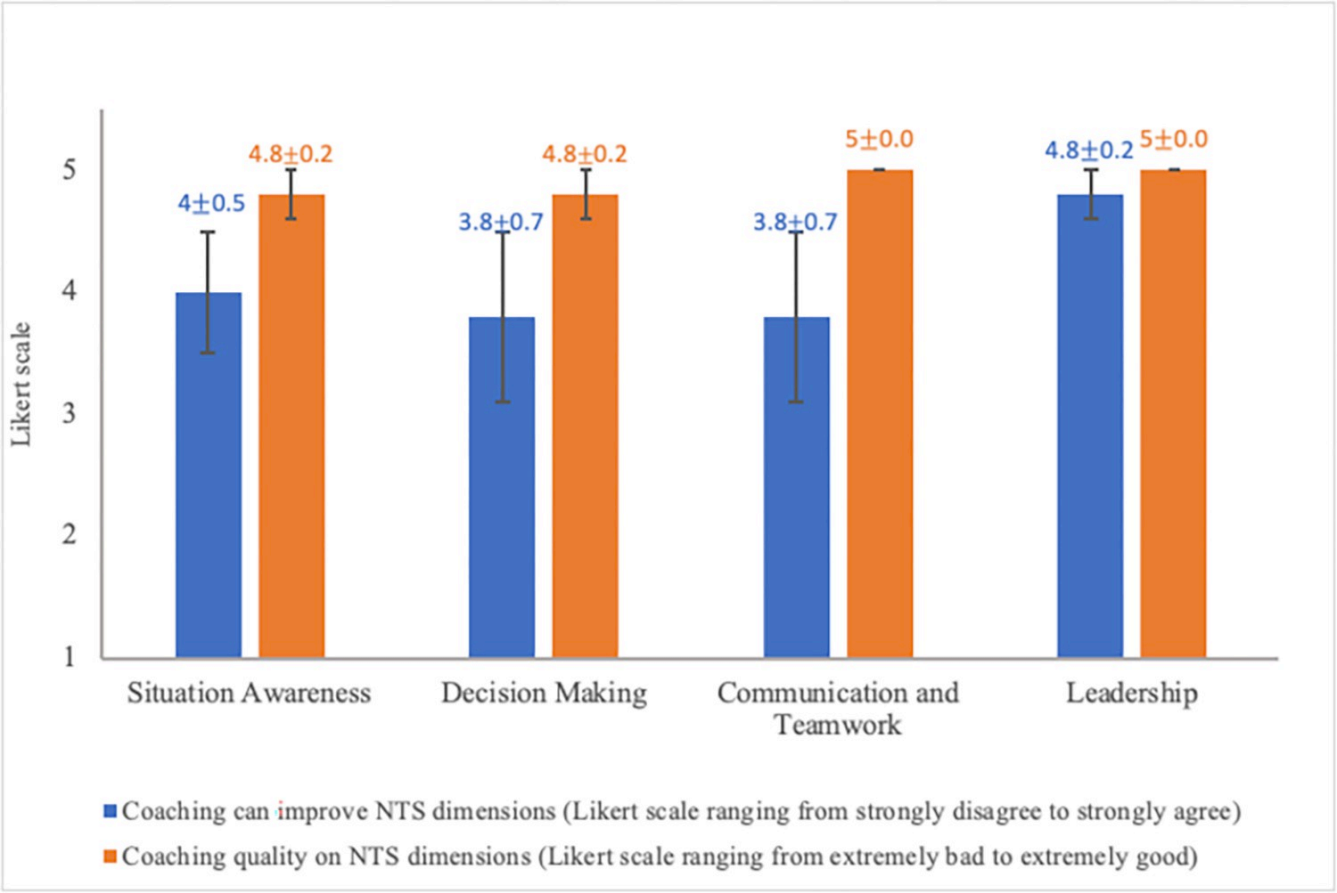
Post-coaching survey responses.

Examples of exemplar and non-exemplar behaviors identified by the expert raters during assessment of the surgeons’ NTS are in S1 Table. Fig 3 shows the mean scores from the survey questions. Error bars represent SEM. For surgeon satisfaction, surgeons believed the coaching sessions could improve their leadership the most (4.8±0.2). Overall, surgeons rated the coaching quality on all NTS dimensions very highly. For learning outcomes, there were mixed reactions from surgeons as to whether the coaching could improve their NTS during the intervention. While decision making and communication and teamwork had a neutral reaction (3.8±0.7), situation awareness (4.0±0.5) and leadership (4.8±0.2) had a stronger positive reaction (Fig 3).

Table 3 shows the mean and SEM of surgeons’ NTS before and after they received the coaching, as well as the effects size of the coaching intervention. Coaching had a medium-to-large effect on situation awareness (*d* = .65), medium effect on leadership (*d* = .41), small effect on communication and teamwork (*d* = .14), and no effect on decision making (*d* = 0). Overall, the coaching intervention had a small-to-medium effect on total NTS (*d* = .33).

**Table 3:** Mean, SEM scores, and effect sizes for NOTSS categories.

NOTSS category	Mean (SEM) score		Effect size (Cohen’s *d*)
	Before coaching	After coaching	
Situation awareness	3.53 (.22)	3.93 (.07)	.65
Decision making	3.93 (.07)	3.93 (.07)	.00
Communication and teamwork	3.67 (.13)	3.73 (.12)	.14
Leadership	3.53 (.22)	3.80 (.11)	.41
Total NTS	3.65 (.08)	3.83 (.05)	.33

## Discussion

Leveraging adult learning and self-determination theories, we developed a specialty-agnostic NTS coaching framework for individual coaching sessions with robotic surgeons. The framework was used to deliver NTS coaching sessions to robotic surgeons. The strongest effect sizes were found in surgeons’ situation awareness and leadership skills. The coaching had a small effect size on surgeons’ communication and teamwork and no effect on their decision-making skills. The coaching had small-to-medium effect on surgeons’ overall NTS.

Surgical coaching is a quality improvement method that improves clinical outcomes [43], patient safety [44], and provides opportunities for continuous professional development of practicing surgeons [45]. In this work, we proposed a quality improvement initiative in the form of an NTS coaching program delivered as individual sessions to robotic surgeons. Previous works have delivered NTS coaching to surgeons in group settings where many NTS constructs were covered [46, 47]. These group coaching sessions were shown to improve NTS, compared to individual coaching where surgeons were coached on only one or two NTS constructs [21]. While group NTS coaching sessions offer several advantages, there are potential limitations that should be considered. First, surgeon participants in a group coaching session may have varying levels of NTS proficiency. Hence, tailoring the coaching content to meet the needs of all participants may be challenging. Second, some surgeons may be hesitant to openly discuss personal challenges, mistakes, or self-identified areas of improvement in a group setting due to confidentiality and social desirability biases. Such reluctance can hinder the depth of discussions and limit the effectiveness of the coaching session. Third, group dynamics can be influenced by dominant biases such that surgeons with stronger personalities may monopolize discussions, limiting the participation of quieter surgeons. This can hinder the coach’s ability to engage all surgeons effectively. To mitigate some of these limitations, we proposed a framework for individual surgical coaching that addresses all NTS constructs and is targeted and personalized to each surgeon based on a needs assessment. Educationally significant effect sizes (*d* = 0.33–0.65) were gotten after applying the framework in an NTS coaching program, further alluding to the impact of this quality improvement initiative.

This study involved a “trial-and-learning” approach, similar to the Plan-Do-Study-Act (PDSA) quality improvement approach for rapid cycle improvement in healthcare [48]. In our approach, a suggested solution for improvement (NTS coaching program) was made and first tested on a small scale (five surgeons) before any large-scale changes can be made [49]. The study phase of PDSA allows for investigating what went right, what went wrong, and what needs to be changed. These “lessons learned” are then incorporated into the next cycle of improvement. In our NTS pilot program, we observed some things that worked well. First, there was a dedicated coordinator who was involved in surgeon recruitment, performing observations, coordinating expert raters, delivering the coaching sessions, and following up with observations. This helped to improve the structure and organization of the program. Additionally, the participating surgeons provided high levels of engagement and support during the program. Finally, as a single-institution program, we received support from leadership to implement the coaching program. This indicates an alignment of the NTS program with the healthcare system’s goals and commitment to continuous improvement. We also identified a few challenges with the program. Since surgeons were only assessed on very specific events and time windows during RAS, we had to extract these periods from the video recordings. Additionally, to improve audibility of the recordings, the video files were synchronized with the microphone recordings. Hence, it took significant time and effort to conduct these audio and video editing. Expert-rater assessments required significant time and efforts to identify exemplar and non-exemplar behaviors of each surgeon. Finally, a major recruitment criterion was that surgeons perform at least one robotic procedure every two weeks. However, we still experienced situations where robotic cases on a surgeon’s schedule was limited when an observation was due.

In this work, summaries of exemplar and non-exemplar NTS behaviors observed during the pre-intervention phase were documented. A main cause of non-exemplar situation awareness behavior was a lack of awareness of the environment. Both individual and shared situation awareness among team members is critical in ensuring safe surgeries [50]. A surgeon’s ability to proactively anticipate potential issues will allow for preventive measures and prompt response to emerging challenges [51]. Non-exemplar behaviors related to communication and teamwork involved the surgeon giving instructions without a specific receiver’s name mentioned, not adopting checkback and close loop communication strategies, and making incomplete requests. Research has shown that nearly 60% of communication disruptions in RAS are attributed to repeat communications either because the message was heard by the receiver or there was no acknowledgment from the receiver [10]. Exemplar decision making behaviors identified in this work were mostly related to technical skills. A surgeon’s decision-making skills can impact technical proficiency by guiding the choice of surgical techniques, instrument selection, and overall strategy. Non-exemplar leadership behaviors were related to inadequately setting and maintaining standards in the OR, especially during the surgical timeout. The surgical timeout is an essential component of patient safety in the operating room, done with the surgical safety checklist [52]. Inadequate or absent timeout was reported as one of the most common causes of sentinel surgical events [53]. One study showed that clinicians believe leadership of the surgical safety checklist be primarily taken by the surgeon [54].

To better understand the mixed reactions from the learning outcomes, the performance improvement outcome was measured. In this work, the post-intervention NTS assessment scores of situation awareness and leadership improved, compared to the pre-intervention assessment scores while the scores remained relatively unchanged for communication and teamwork and decision making. The findings from the performance improvement measures align with those from the learning outcomes measures. This alignment suggests that the coaching content was successfully translated into real-world surgical practice, demonstrating the impact of the intervention on surgeons’ abilities.

This study is not without limitations. The NTS coaching program was piloted with a small sample of five surgeons. Hence, we were unable to run any statistical significance tests to determine if the coaching indeed improved NTS. However, the coaching intervention had educationally significant effect size on some NTS constructs. Given the prospects of this work, future studies will focus on expanding the coaching program to more surgeons. Also, this was a pre-post study and might have limited the assessment of the framework’s effectiveness. Future work will focus on estimating the NTS retention of surgeons after coaching (i.e., more follow-up observations) to ascertain if the improvement was sustained. In addition, the NOTSS scores and coaching material were a direct result of subjective expert raters’ assessments. Behavioral marker systems like NOTSS are the current gold standard for NTS assessment in surgeries and can be subjective and sometimes, ambiguous. To mitigate rater biases, we utilized consensus meetings. Other researchers have utilized this approach in other healthcare domains to discuss and reconcile any discrepancies in raters’ assessments [55, 56]. More work is needed to develop objective and data-driven NTS assessment approaches. Finally, we recognize the potential Hawthorne effect in our study, but given the high-pressure surgical environment, it’s unlikely surgeons consciously altered their behavior due to their awareness of the video recordings. The critical nature of the surgical demands on surgeons likely minimized any such effect on our findings.

## Conclusion

In this paper, we presented a quality improvement initiative to enhance the NTS of robotic surgeons through the implementation of a coaching program that leverages the proposed NTS framework. Recognizing the importance of NTS in RAS, our initiative shows a commitment to continuous improvement of patient safety and quality of care.

## Supporting information

S1 Table Summary of exemplar and non-exemplar behaviors identified during NTS assessment. (DOCX)
