# Supplementary material for: Development and application of a non-technical skills coaching intervention framework for surgeons: A pilot quality improvement initiative
Source: PLoS One. 2024 Nov 8;19(11):e0312125. doi: 10.1371/journal.pone.0312125 (PMC11548760; doi:10.1371/journal.pone.0312125)
Supplement: S1 Table — (DOCX) [file pone.0312125.s001.docx]

**Supplementary Material Table 2. Summary of exemplar and non-exemplar behaviors identified using the NTS framework**

| Category | Element | Example of behaviors | |
| --- | --- | --- | --- |
|  |  | Exemplar | Non-exemplar |
| Situation awareness | Gathering information | 1. Optimize lighting and physical ergonomics  2. Check current operative findings for patient safety before proceeding to next step | Low environmental situation awareness (unawareness of a loss of insufflation and who is at the other end of dual console) |
|  | Understanding information | Understand:  1. Orogastric tube was not working well  2. Effect of wrong trocar placement on patient safety and subsequent robot docking  3. a consistent reduction in belly space means insufflation is being lost  4. the balance between teaching the trainee and maintaining patient safety |  |
|  | Projecting and anticipating future states | Anticipate needing specific instruments depending on outcome of current operative finding | Failure to anticipate:  1. Surgeon, monitor, and lights are in the way of boom while driving robot for docking  2. Docking by scrub and resident unsupervised leading to a wrong grasper inserted to robot’s arms  3. No footboard before positioning bed in reverse Trendelenburg and how unsafe that is for patient |
| Decision making | Considering options | 1. Consider surgical techniques with assistant  2. Consider options of specific equipment or material needed with scrub  3. Specifically seeks opinion from other team member when unclear  4. Generate different possibilities (to pair or not pair patient bed with robot) during robot docking |  |
|  | Selecting and communicating options | 1. Communicate to the team progress of procedure  2. Recognizes and articulates problems with the Nathanson retractor and seeks help from equipment rep​ in the OR  3. Communicate thought process to assistant when docking robot  4. Discuss options with assistant and make provisions for plan “B”​ |  |
|  | Implementing and reviewing decisions | 1. Review incision site after initial decision  2. Review priority of need to anesthesiologist (orogastric tube vs bed position in reverse Trendelenburg) by triage  3. Review a step made during docking and asks equipment rep if that was correct  4. Double checks for hiatal hernia from another angle ​ | Calls for external help from a specific person outside OR without explaining to rest of team why that person; team members can perceive that as a lack of faith in themselves and/or attending. |
| Communication and teamwork | Exchanging information | 1. Strike a timely balance between letting fellow take autonomy but also providing help  2. Check back and close loop with scrub while returning instruments especially sharp objects  3. Address team members by name  4. Gives very specific instructions without room for ambiguity (e.g., exact bed angle and orientation; what grasper goes into which robot's arms) | 1. Gives instructions without receiver  2. No checkbacks and close communication loops  3. Makes requests that are incomplete |
|  | Establishing a shared understanding | Establish shared understanding with:  1. Scrub on materials taken out of the patient body, and specific tasks to be done when surgeon is on-console  2. Assistant on how to insert trocar  3. Rest of team by informing them of major milestones in procedure |  |
|  | Coordinating team activities | Coordinate:  1. team-based activities like robot driving and docking  2. with trainee to figure out landmark for locale, incision, and trocar placement​ | Failure to provide cue to rest of team to indicate the completion of robot teleoperation and subsequently, an immediate need for the team to coordinate and undocking the robot |
| Leadership | Setting and maintaining standards | During timeout,  1. initiate timeout, introduced self and role, focus on active listening and stopped working | No surgeon safety statement and no team introductions during timeout |
|  | Supporting others | Supports assistant and other members of the team |  |
|  | Coping with pressure | Remain calm:  1. even though gas tubing was off and insufflation was getting lost often  2. even after making multiple repeat requests to other members of the team  3. while experiencing difficulty docking a single-port robot |  |
